# Unexpected Effects on Some Spanish Cultural Landscapes of the Mediterranean Diet

**DOI:** 10.3390/ijerph18073829

**Published:** 2021-04-06

**Authors:** Pedro Tomé

**Affiliations:** ILLA-CSIC, 28037 Madrid, Spain; pedro.tome@csic.es

**Keywords:** Mediterranean Diet, Mediterranean landscapes, environment, sustainability, agro-industrial landscapes

## Abstract

The declaration of the Mediterranean Diet as Intangible Cultural Heritage of Humanity in order to preserve a cultural and gastronomical legacy included the protection of lifestyles, knowledge, sociability, and environmental relationships. However, the patrimonialization, popularization, and globalization of a certain conception of this diet have turned it into a de-territorialized global phenomenon. As a consequence of this process, it has been necessary to notably increase the production of its ingredients to satisfy its growing demand, which, in turn, has generated “secondary effects” in some Mediterranean environments of Southeastern Spain. If, on the one hand, their wealth has increased and population has been established, on the other hand, the continuity of certain cultural landscapes linked to local knowledge and particular lifestyles has been broken, replacing them with agro-industrial landscapes exclusively at the service of production. This, at the same time, has caused social and environmental inequalities

## 1. Introduction

It has become commonplace to bring up a certain saying attributed to Josep Pla—“the cuisine of a country is its landscape set in a pot”—to refer to the relationship between diet and landscape. As if, by necessity, the landscape of a particular space limited and conditioned its dominant diet, establishing a relationship of dependence between environmental conditions and the possibilities of nourishment. In a way, this long-held idea underlies, as well, the Resolution approved by the European Parliament [1] on 12 March 2014, on “European gastronomic heritage: cultural and educational aspects (2013/2181(INI))”. This resolution, not only contemplating previously approved documents by other institutions, but also the UNESCO Convention for the Safeguarding of the Intangible Cultural Heritage (2003), as well as the inclusion of the gastronomic meal of the French and the Mediterranean Diet in the UNESCO Representative List of the Intangible Cultural Heritage of Humanity, explicitly links this gastronomic heritage to landscapes. It states that UNESCO’s acknowledgment of the Mediterranean Diet requires the “promotion and establishment of patterns of behavior that ensure a healthy lifestyle thanks to a holistic approach that takes into account aspects relating to education, food, school, family life, nutrition, territory, landscape, etc.”, the resolution contends that “the European heritage is made up of a set of tangible and intangible elements and, in the case of gastronomy and food, is also formed by the locality and landscape from which the products for consumption originate”. For this reason, the resolution continues, on top of emphasizing the diversity of regions and landscapes, it invites the support initiatives “to promote and preserve all the territories, landscapes and products that make up their local gastronomic heritage”.

In this paper, I present some concrete examples of the environmental, social, or cultural impacts that the rise of this diet is having, or at least a certain way of conceiving it, in some specific areas of the Spanish Mediterranean. Due to the mobility restrictions inherent to the current pandemic, these pages cannot include recent information produced by field work and are based on the analysis of the existing literature on these places, completed with a follow-up of the documentation generated by the administrations public and collected in the specialized magazines. Thus, in the first place, I tell how the conception of the Mediterranean Diet promoted by UNESCO and other organizations has been gradually replaced by a biomedical consideration that, instead of emphasizing landscapes and lifestyles, highlights the value of products regardless of their origin and how they were produced. Below are the specific effects that this conception is having on certain cultural landscapes that are being replaced by new agro-industrial landscapes that, in turn, are changing the ways of life that the UNESCO declaration said must be protected. With this, it is exposed how the declaration of the Mediterranean Diet as a World Heritage Site and its promotion, in addition to the positive effects on the health of those who consume it, may also have had negative effects in some environmental and social contexts.

## 2. From Particular Mediterranean Diets to a Global Diet

The intimate connection between landscape and diet was already questioned in 1985, by Sidney Mintz [2], when analyzing the role of sugar in modern history in *Sweetness and Power*. He states that, generally speaking, human groups hardly ever eat every foodstuff found in their surroundings, and, while some are cherished, others are rejected. Likewise, Jesús Contreras, Antoni Riera, and F. Xavier Medina [3] have pointed out that up-keeping this model of relationship between landscape and diet supposes a relapse into geographical or environmental determinism that ignores the incidence of exchange and other social and cultural processes that take place on every location. For this reason, they consider it that in practice “a country’s cuisine is the products in its markets, set in a pot” [4] (p. 18). From this point of view, the offer of food supply and the preferences about it would be what shapes what is eaten, rather than ecological conditions. In this sense, if foodstuff turned into goods available anywhere in the world, then the link between nourishment and landscape becomes a secondary one. In fact, it is not unusual for consumers to be unaware or uninterested in the geographical origin of most of the food they consume (see Barreiro [5], p. 12). This, no matter how the succession of crises in the food supply chain (plagues in plants, animal disease, etc.), is favoring a trend toward the consumption of “local products” that can be partly tied to a need to “see and feel the origin of what is eaten” [6] (p. 46), and partly, to other factors, such as nostalgia, or culinary [7] or consumption-based nationalism [8]. Or it could be leading to a combination of all of these, as seen in the campaign “The richest country in the world”, developed by the Ministry of Agriculture, Fisheries and Food to promote “Spanish food products”. In this campaign, a series of food-based moving “still lifes” recreate national landscapes intending to awake “deep feelings” on sensing “the greatness and diversity of our products”, and “presenting a positive and proud vision on our food products” (https://www.alimentosdespana.es/es/campanas/ultimas-campanas/alimentos-de-espana/el-pais-mas-rico-del-mundo/default.aspx (accessed on 29 January 2021)).

However, if the relationship between landscape, product, and diet is no longer a direct one, but one that comes mediated by other elements (markets, ideologies, cultural values, etc.), it is worth questioning if the foodstuff that include in their nomenclature the name of a place must have been produced fully in said location or if it is enough for this place to be present in some phase of its manufacturing. For instance, when making wine coming from any of the Designation of Origin (DO) areas, must the grapes have been harvested in said DO, or can they have been harvested far away and processed in the DO? Or, considered from across the pond, can we consider a “Mediterranean diet” one that employs products with similar characteristics and features to those of the heterogeneous Mediterranean landscapes when these products have been grown in their totality in California or Mexico? Ultimately, does a Mediterranean Diet require a Mediterranean landscape? Although, apparently, the answer to these questions seems simple, in practice it is conditioned by the existence of different conceptions about what the Mediterranean Diet is.

As part of the defense prepared by the permanent representatives of Spain, Greece, Italy, and Morocco to the UNESCO to promote the inscription of the Mediterranean Diet in the Representative List of the Intangible Cultural Heritage of Humanity, it is explicitly stated that “The Mediterranean Diet is the set of skills, knowledge, rituals, symbols and traditions, ranging from the landscape to the table, which in the Mediterranean basin concerns the crops, harvesting, picking, fishing, animal husbandry, conservation, processing, cooking, and particularly sharing and consuming the cuisine” [9] (p. 6), so it cannot be separated from some “Cultural functions” based “on the symbolic and ethical relationship our communities have forged with nature, landscapes, seasonal cycles and the sustainable management of natural resources, particularly water” [9] (p. 7). From this point of view, the Mediterranean Diet would be “a transversal element, from the landscape to the table, a prominent example of the links between intangible and tangible heritages and the cultural, historical and identity references embedded in our communities” [9] (p. 8). This would also be justified by the fact that “the progress has been made on the legislative front to label and protect landscapes, cultural spaces and artisanal, traditional and local productions, while promoting their rural communities Research institutions, universities and foundations are involved in this effort and continually support the advancement in training, in the safeguarding of landscapes and biological diversity, in the sustainable use of natural resources and in the promotion of traditional techniques and, above all, innovation related to the element” [9] (pp. 10–11).

However, in spite of these considerations, those engaging the information offered by the UNESCO in regards to the 2013 inscription of the Mediterranean Diet in the Representative List of the Intangible Cultural Heritage of Humanity (https://ich.unesco.org/es/RL/la-dieta-mediterranea-00884 (accessed on 22 January 2021)) will be able to confirm how, even though this list includes knowledge, skills, traditions, agriculture-related symbols, ways of managing food, commensality, fellowship, communal living, intercultural dialogue, craftsmanship, holidays, markets, etc., there is no reference to Mediterranean landscapes. Likewise, any similar mention is missing from the inventory that supports the Intangilbe Cultural Heritage candidacy presented by Spain (https://ich.unesco.org/doc/src/19700.pdf (accessed on 22 January 2021)). Landscape also fails to play a fundamental part in the candidacy of the set of institutions who joined the proposal, whose consent is included in https://ich.unesco.org/doc/src/16813.pdf (accessed on 22 January 2021). In fact, half of the associations or institutions that supported this candidacy present it both generically and vacuously, as an indispensable addendum that needs to be named in order to fulfil bureaucratic requirements. Furthermore, references to the landscape appears in a vague text—the same one in almost every case, merely changing the name of the signing institution—that states the inclusion of the Mediterranean Diet in the aforementioned list must be contemplated due to the negative effects that “globalization and socio-cultural changes” are having on “the health and welfare of people, the stability of the rural population, and the protection of the environment, landscape and culture of Mediterranean communities” (cf. in said document, e.g., Spanish Association of Olive Tree Municipalities/Asociación Española de Municipios del Olivo and other 25 associations and institutions). At the same time, a significant number of the signers do not even explicitly mention the relationship between landscape and diet, or they present a variation on the stated formula.

Something similar takes place with the 41 proves of consent by the associations or institutions for their knowledge, practices, or activities to be disseminated in order to support the presentation of the candidacy of the Mediterranean Diet to UNESCO, linked to the city and province of Soria. While most of them mention landscape in passing, only the document signed by the Sorian Association for the Defence and Study of Nature—Asociación Soriana para la Defensa y Estudio de la Naturaleza ASDEN (Ecologistas en acción)—points out the importance of this inclusion, “in order to better the health of citizens, as well as their quality of life, collaborating with the conservation of peculiar ecosystems with centennial traditions, and the conservation of peculiar landscapes” (https://ich.unesco.org/doc/src/16813.pdf (accessed on 22 January 2021). Annexe 4).

In spite of its continental features and its lack of proximity to the Mediterranean Sea, Soria’s shows of consent proved decisive in the process of making the Mediterranean Diet Intangible Heritage, since Soria was the first Spanish town to support the candidacy through a long process where diverse interest came into conjunction [10]. During the undertaking of fieldwork in Soria, Consuelo Álvarez established how spontaneously her interviewees identified Mediterranean Diet and olive oil. For this reason, she remarks, “returning to the Mediterranean diet means returning to olive oil. The symbolic function of this oil is its meaning, beyond any dietary qualities, pertaining to imagined virtues” [10] (p. 419). The fact that olive oil can contribute to the creation of an “imaginary of Soria washed by the Mediterranean Sea” [10] (p. 413) showcases how landscape becomes something accessory for the diet, since the totality of Soria province finds itself beyond the reach of the area of olive tree production in the Iberian Peninsula (https://www.mapa.gob.es/app/MaterialVegetal/fichaMaterialVegetal.aspx?idFicha=6 (accessed on 18 January 2021)). That is, apparently, the Sorian support for the Mediterranean Diet would assume that one of the defining elements of a local diet—every association endorsing the project showed the connection between their local or provincial activities and this local diet—is a product that requires importation from other places, since the province’s environmental conditions preclude its manufacture. In this case, therefore, the knowledge, traditions, symbols, food management, etc., presented by the UNESCO declaration are necessarily extra-local and, in consequence, point to a disconnection between diet and landscape.

This separation becomes possible because in the particular case of the Mediterranean Diet, this crucial role in the patrimonialization process has been played by agents linked in one way or the other to the medical profession and food science. Within the 53 backers of the inscription of the Mediterranean Diet presented by the permanent representatives of Spain, Greece, Italy, and Morocco, we find, among others, the Spanish Academy of Nutrition and Food Science, the University of Barcelona’s Research Group for Communitarian Nutrition (Grupo de Recerca en Nutrició Comunitària), the Spanish Federation of Food and Wine Guilds, the Mediterranean Diet Foundation, the Mediterranean Agronomical Institute of Zaragoza, the Department of “Food Systems, Culture and Society” of the Open University of Catalonia, the NGO Nutrition without Borders, Foundations for Nutritional Research, the UNITWIN-UNESCO Chair of Research, Planning and Development of Local Sys-tems of health of the University of Las Palmas de Gran Canaria, the UNESCO Chair of Visual Health and Development of the UNESCO chairs network of the Polytechnic University of Catalonia, the Foundation for the Promotion of health, etc.

In consequence, its relevance is supported by the positive effects following this diet has, and not on its connection with social or cultural processes in any particular region. However, as an “ideal model of a healthy diet” [11] (p. 19), some of its particular elements can be replaced for some undisruptive alternatives, that “reinterpret” a diet that can be consumed anywhere in the world. Moreover, as an ideal “it becomes more cohesive and common the further away we move from the Mediterranean; so much that it would not be uncalled for to wonder if, in fact, this diet is not really a North American or Australian creation” [12] (p. 175). In any case, the expansion of the model and its reproduction as an ideal removed from the Mediterranean has become possible largely because a selection or pyramid-like hierarchy of its components based on their health benefits [13] had already been produced through bio-medical discourse. Said discourse, by situating food products—and in consequence, how to engage them—at the center of the diet, allows their replacements by others that occupy the same place in the “pyramid” and that are produced locally to the place of consumption. Even when this place is situated far away from any Mediterranean landscape.

If there ever was such a thing as the customs, styles, and values that classical social anthropology in the 20th century identified as prototypically Mediterranean in regards to food consumption, the actual identification of the Mediterranean Diet and its bio-medical justification has superseded them for good. This process involves a “substitution of traditional and socially regulated reasons that recommended eating this or that, in a particular state, for other nutritional and scientifically legitimated reasons, in such a way that the hierarchy of goals of the act of eating—pleasure, socialization, health—varies” [14] (p. 201). Paradoxically, a process of turning diet into heritage, supposedly intended to protect “local” and “traditional” lifestyles characterized by particular models of sociability sees them now taking a second place in the face of “medical rationality” [14] that turns health into the fundamental social value social life must revolve around. At the same time, the inclusion of the Mediterranean Diet in a context of biopower characterized by the medicalization of food disregards the all the other elements of that sociability, including socio-environmental contexts, that gave it a particular sense. On the contrary, by replacing this pattern by a set of segmented units, the connection between the different components of this lifestyle breaks apart and these components stop making sense in the local sense, to become understandable only from a global point.

In this setting, it is not surprising, for instance, that the hundreds of cruise ships that would, before the emergence of this devastating pandemic, cross the waters of the Mediterranean, carrying thousands of tourists from port to port, could offer in their many restaurants “Mediterranean gastronomy” or “Mediterranean Diet” (https://cruceroland.com/recetas-sanas-faciles-gastronomia-mediterranea/ (accessed on 20 January 2021)), while both the ecosystems and coast-adjacent residents see themselves as being highly affected by the environmental—among other kinds—pollution these ships generate. In fact, even though it is difficult to establish a linear causal relationship, the emphasis on the products without a focus on the environmental contexts where they are grown sometimes generates very negative effects on the second of these.

## 3. New Agro-Industrial Landscapes and Local Knowledge

Political–cultural ecology, proposed by Eric Wolf [15] to develop some aspects of classical cultural ecology of Steward [16] and has subsequently been made explicit in numerous works in different senses [17,18,19,20], has revealed how complex processes operate that lead to the selection of certain spaces as protected or conserved areas, compared to others that must be used for production or other diverse uses. This changing spatial categorization, which can be induced by agents internal or external to the landscape itself, shows that landscapes include not only natural relationships, but also social, political, economic, and ultimately cultural relationships. This idea, which led Carl Sauer to propose the concept of “cultural landscape” in 1925, —“the cultural landscape is fashioned out of a natural landscape by a culture group. Culture is the agent, the natural area is the medium, the cultural landscape the result. Under the influence of a given culture, itself changing through time, the landscape undergoes development, passing through phases, and probably reaching ultimately the end of its cycle of development. Whit the introduction of a different, that is, alien culture, a rejuvenation of the cultural landscape sets in, or a new landscape is superimposed on remnants of an older one”—[21] supposes that what we call natural landscape is transformed by the action of human groups into a cultural landscape. Since Sauer proposed this concept, social anthropology and cultural geography, as well as other social Sciences, have converged in the analysis of the interrelationships between natural and cultural processes using it [22,23,24,25,26,27,28,29,30,31,32,33]. Therefore, to understand landscapes, it is necessary to integrate, together with the “natural” elements, the multiple perceptions, representations, and symbols that build them, because any landscape, since it is culturally invested, is both material and immaterial, tangible and intangible. In any case, that the landscape can be understood as a cultural construction should not mean that it is a mere “text”, a “narration” from which the material elements disappear [34]. Therefore, any cultural landscape impregnated with relationships becomes a social construct. The publication of “Without nature, without culture. The Haguen case” by M. Strathern [35] consolidated the need to conceive nature and the natural, culture and the cultural, as social constructions and not as given entities. Although there are multiple ways of understanding the social construction of nature, social anthropology and much of the cultural geography of recent decades has assumed this position as unquestionable [36,37,38,39,40,41,42,43,44,45,46,47,48,49,50,]. However, it should not be forgotten that, because they are social constructions, “patters of authority are therefore inscribed in landscapes and reflected in ecological pattern and process: physical spaces and biophysical features become socialized and institutionalized over time, and localities are produced through the institutional and political interconnections across space and time.” [51]. Thus, the transformation of landscapes, which inevitably includes transformations of social relations and everything associates with the social system, shows the continuous reconfiguration of the geographic bases of political power [52].

### 3.1. Socio-Environmental Transformations

These processes are notorious in some places that are radically transforming their productive activity and their socio-environmental relationships to satisfy the global demand for products that are in the base of the Mediterranean Diet. For example, the medical beatification of olive oil and its subsequent identification as a fundamental element of the Mediterranean Diet, joined to other related factors, has necessitated an increase of oil production. In the Spanish case, this has been achieved in two different ways: by expanding the area dedicated to olive groves [53] and by watering the existing ones. Furthermore, *Olimerca* [54] a magazine aimed at the olive oil market and sector, using data from the Ministry of Agriculture, informed that “olive plantations in Spain have not stopped growing in 2019, and throughout the year 99 hectares have been planted each day, (…) making up for a 4% increase in the last five years (since 2014)”. This growth is making an increasing number of Andalusian municipalities depend on single-crop farming, with all the related risks and effects. At the same time, the increase of olive grove watering—notwithstanding some voices that defend its sustainability—is causing an over-exploitation of water resources, both underground and aboveground. The Guadalquivir Basin Authority (Confederación Hidrográfica del Guadalquivir), the official organ in charge of the management of the basin, has pointed out recently that every water-consumption plan drafted in the past twenty years was off, since “the reality of the demand had changed; it was vastly superior to the forecasts and was directed mostly to olive grove drip irrigation, primarily with unregulated or underground water. (…) This demand was absolutely untenable and threatened the very possibility of an effective management of the basin and its legally consolidated uses, already undergoing a chronic deficit and had extremely serious effects on the base flow and the water level of numerous brooks and springs. In addition, the large olive grove extension created a potentially infinite demand” [55] (p. 11). On the other hand, even though this process has supposed important progress in rural areas, contributing to the retainment of the population in marginal zones and preventing a rural exodus akin to the one suffered in other Spanish regions, this growth comes with its costs: the decrease of water volume in many of the aquifers of the basin [56] (p. 21). In other words, the increased demand of olive oil, due to a great extent to the success of a bio-medically underpinned Mediterranean Diet, is quickly changing a landscape that is turning industrial by leaps and bounds.

Furthermore, of a drastic nature are the changes generated in the landscape by the importance the Mediterranean Diet gives to horticulture. According to the World Vegetable Map 2018 report, developed by RaboResearch Food & Agribusiness, there is a “growing importance of production in greenhouses and vertical farms” [57]. According to this report, out of the half a million of hectares occupied by greenhouses in the world, 30% would be distributed between China—82,000 hectares—and Spain—70,000 hectares. A glossed analysis of the report’s data shows the fast growth of greenhouse fruit and vegetable production in all of the Mediterranean basin: Only Almeria province, in the Spanish Southeast, has almost as many greenhouses as both South and North America. Other Mediterranean countries also have seen a notable greenhouse expansion—both plastic and glass—on their soil: Italy (42,800 hectares), Turkey (41,300), Morocco (20,000), France (11,500), Israel (11,000), and Egypt (6800). In the Spanish case, a single town, El Ejido, in Almeria province, counts with some 13,000 hectares taken by greenhouses, whose plastics end up partially in uncontrolled dumps once they stop being of use. The real dimensions of this occupation becomes apparent by noting that, in the whole of South America, greenhouses do not make up a full 14,000 hectares. The local avifauna is the first to be affected by the growth of this “plastic sea”, as the Almerian West is known, since most of its plants are undercover. Due to this, the whole ecosystem is being affected in one way or the other. If it is true that every landscape continuously shifts, as dynamism is one of its characteristics, in the case of El Ejido, a “rupture of consistency” [58] (p. 31) has taken place in the modeling of the landscape. In particular, a specific type of Mediterranean landscape, the product of the use of grass for semi-nomadic cattle in winter in combination with the production of barley and prickly pear, has been radically replaced for a completely new one, particular to an agro-industrial model based on a social and environmental imbalance. Moreover, a break with the processes of long-term change that had characterized the landscape previously has allowed for one of the driest regions of Spain to become, as a regional politician would say, “Europe’s vegetable garden”. However, as Kearny [59] noted in 1996, with respect to the expansion of the commercial farming model in Northwest Mexico, California, Oregon, and Washington, it has replaced local landscapes with a uniform one.

The price paid in exchange has been an increase in social inequality up to hard-to-conceive levels and the depletion of aquifers that the greenhouse owners want to recover by expanding saltwater desalination and reducing underground water consumption [60]. In the case of the Region of Murcia, bordering with the aforementioned province, vegetable irrigation is guaranteed through the existence of historical water transfers that guide the liquid through miles-long aqueducts (the water transfer from the Tagus to the Segura River, the most important of the several transfers to be found on the Spanish basin of the Mediterranean, measures almost 250 km (155 m) long), causing the perverse environmental effects of taking the water where the production is, as opposed to taking the production where the water is, to be felt quite far away from the place that most benefits from them from an economic standpoint. Certainly, it is not possible to establish a direct cause–effect relation between the Mediterranean Diet heritage status and the landscape transformations of the Iberian Peninsula’s Southwest: initiated beforehand and responding to multiple factors. However, it is apparent that the Mediterranean landscapes that produce the goods that comprise this diet—at least in the Spanish case—are being subjected to a type of agro-industrial pressure that, it seems, should be incompatible with the effects sought after by the UNESCO.

### 3.2. The Breakdown of Particular Ethno-Ecologies

On top of the substitution of heterogeneous rural landscapes associated with local production by others, homogenous and agro-industrial in nature, the emphasis on the product itself can also affect the ethno-ecologies and put “bio-cultural memory” at risk [61]. As these authors show, the industrial management of agriculture, joined to the incentives other economic sectors use in order to motivate an abandonment of traditional agriculture in the island of Majorca, produces a “loss of the memory associated to traditional and artisanal skills, more linked to traditional feminine agricultural roles” [61] (p. 499). In this particular case, these researchers prove how the knowledge tied to local varieties of tomatoes—on which their work focused—is maintained thanks to the partial support for agroecology of scarce excess, linked to self-supply, developed in the margins of professional agriculture. However, even though these groups—the retired who hold traditional know-how or young people who “for questions of attachment to the land maintain certain activities related to tradition” [61] (p. 499)—are able to preserve local knowledge related to production, knowledge concerning post-harvest and conservation skills (such as the stringing of tomatoes and their preservation during winter) finds itself in high risk of being lost.

In other occasions, these risks to the upkeep of local know-how is related to the attempts to achieve heritage status for a particular product through a certification of quality. Thus, Cantero and Ruiz Ballesteros [62] counter the institutional certification undertaken by local producer of “rosao” tomatoes in the Sierra de Aracena—a certification previously followed by Ibérico ham producers in the same area in order to obtain the designation of origin of said consumable—with an alternative one. In contrast to the case of the tomato, the Ibérico ham’s status was subordinated to market logics. That is, it was focused “almost exclusively on increasing its market value, leaving behind its domestic and cultural dimensions, and all of this—in itself—is at risk of becoming an environmental and economic issue. Effectively, the over-exploitation of the grassland brings with itself a noteworthy degradation of the ecosystem, and the excessive production, with no quality controls, has caused a serious crisis for the sector” [62] (p. 404). As a similar example, a study on the production of legumes in a region in the center of the Peninsula alerts us of the transformations, both ethno-ecological and undergone by historical social relations, built as the consequence of a productive homogenization that has reduced, in practice, the eight varieties of bean (“judía”) that used to be grown in the region to only one. This productive uniformity in the area of El Barco de Avila was preceded by the intervention of the Protective Geographic Indication (Indicación Geográfica Protegida, PGL) Office, who distributed for several years the same type of certified seeds to all the farmers [63] (p. 252). In spite of this, even though the PGL has simultaneously promoted mechanisms that allow to incorporate an economic value to the bean and the conditions to achieve heritage status, there is still a part of the production circulating in the margins of these channels, linked to a “lifestyle [that] still uses an ethno-ecology that hardly can be accommodated to this institutionalization-derived homogeneity” [63] (p. 250). In fact, it could be suggested that one of the characteristics of industrial agriculture is the abandonment of local knowledge, without considering the multiple connections that may exist between it and technical knowledge [64], as well as the contributions that local knowledge makes—particularly in times of political, economic, and environmental instability or in response to economic, social, or environmental disasters [65] to scientific knowledge. It is not surprising, therefore, that our knowledge of global or local alterations and risks, and the disappearance of particular ethno-ecologies generated by many peoples, can advance at the same speed.

### 3.3. Ecological Agriculture and Agroecology as Alternative Practices

Productive homogeneity allows for the easy identification of a product, and, in consequence, the achievement of heritage status for itself or any other aspect tied to it—an area, a landscape, a custom, a dish, etc. This status, however, may generate discomfort and resistance. For instance, some wineries are abandoning the Rioja Qualified Denomination of Origin (Denominación de Origen Calificada), “in order to offer the consumer the opportunity to discover the diversity of our land” [66] However, diversity can be both a strength and a weakness, as the attempt to create an IGP for Salers cheese, in France: “This managed diversity—the discontinuous, interlinked, and often contradictory local knowledge—is the source of the renowned high quality of the Salers cheese product and a source of power for smaller artisanal producers, yet it also has hindered development of the label as a regulatory instrument, and for somewhat different reasons, the cooperative relations among sector members” [67]. The aforementioned examples show the emergence of new networks “including cattle, microbes, wooden tools, and cheeses, not only people” [37] that make possible alternative visions of the connections between the products that are consumed and the places where they are grown or produced. “Ecological agriculture” and “agro-ecology” are the most reiterated resources in the attempt to regain this unity between landscape and food, since—complying with food-safety-normative and product-traceability requirements—allows consumers to again feel the land in their dishes. The main difference between ecological agriculture and agroecology has to do with the use of local knowledge. While the first refers to environmentally friendly agricultural techniques that do not use chemical inputs produced in laboratories, the second, agroecology, refers to principles developed in particular socio-ecosystems, which include the social aspects of production. That said, the appeal to agroecology is not without its hardships, as the hilarious tales of Chris Stewart in the Granadan Alpujarra reveal, or, alternatively, the difficulties encountered by ecological agriculture in large areas of the Region of Valencia to reduce the dominant monoculture of citrus plants sowing other “ecological” types [68]. In this example, the issue arises that, much like in the case of olive groves, the increase of citrus production (oranges, lemons, tangerines, grapefruits, etc.) has been achieved through watering. Because of this, the waterer communities have improved their systems adding drip irrigation in all of the production; using water that has been treated with chemical fertilizers for the specific use of the common orange—a process known as fertirrigation. “Bio” food regulation explicitly prohibits the use of chemical fertilizers, placing ecological farmers in the position of choosing to remain within the productive standards or to abandon the land, incorporating systems high in alternative risks or assuming risks that are disproportionate.

On the other hand, this move toward the “ecological” or “sustainable” being identified with the “local” or “traditional” may have quite diverse consequences: Some defend this trend as being a result of an exercise in awareness of the limitations of the planet and the effects of climate change; others, however, set their eyes on economic factors and see this as an alternative to the industrial Common Agricultural Policy (CAP) that, they believe, has generated negative consequences in the rural sphere. There can also be found some who understand agro-ecology as a part of the defense of food sovereignty or linked to the love of the land. For others, finally, it is a simple fad that is largely related to “the association of its consumption to a particular status and social class” that turns its products into “referents of exclusivity, sybaritism, and elitism” [69] (p. 67). Indeed, “that there is a complex relationship between class and food consumption is often remarked, first in the obvious sense that particular groups occupy differential market situations in terms of their ability to purchase certain foods, and second in the uses various groups make of foods and food preferences in marking themselves as distinctive from or in some sense like other groups” [70] (p. 773). In this sense, this supposed return to the “local-traditional-authentic”, apparently deriving from the status of heritage of the Mediterranean Diet, cannot be understood disconnected from a system that subordinates culture and environment to a consumption economy guided by the interest of markets, both ethereal and concrete, that have transformed the Mediterranean in a “brand” of indubitable success.

## 4. Conclusions

The conversion of the multiple particular diets associated with the different Mediterranean landscapes in which they were produced and consumed into a single global diet is having very negatives effects on some of the Spanish landscapes associated with the products that medical rationality considers better for health. That is, by focusing the diet on the products, regardless of how and where they are produced, some of the landscapes, far from being protected as the UNESCO declaration foresaw when it was incorporated into the Representative List of the Intangible Cultural Heritage of Humanity, are suffering irreversible transformations that distance them from the complex cultural landscapes stereotypically characterized as “Mediterranean”, becoming new and uniform agro-industrial landscapes. These new landscapes, subordinated to market logics, by breaking with the historical processes that have originated them, produce the “Mediterranean”, but they are no longer “Mediterranean”.

The transformation of cultural landscapes into a commodity destined exclusively to produce merchandise in a process of de-historization that causes an emptying of the meanings linked to local identities and connections, allows us to evoke the image of the Mediterranean within a transnational political economy of feelings [34,71] that paradoxically facilitates a new connection, in the global market, of landscapes and foods: We consume de-historized and de-territorialized Mediterranean landscapes in a diet that, based on products, lacks a temporal and territorial reference.

Naomi Klein, in her renowned work *No Logo*, suggested that “we were so busy analyzing the pictures being projected on the wall to notice that the wall itself had been sold” [72] (p. 161). The same seems to have happened to the Mediterranean, a name that, more than standing in the place of geography, is an evocation, an image that serves multiple goals and accommodates itself neatly to an economy that turns the market of the intangible into one of the main instruments of accumulation of capital. It could be said that, in many ways, the term “Mediterranean”, both in relation to food and landscapes, customs, etc., has come to be a heritage brand that allows for the possibility of a certain success in market terms and that, simultaneously, comes into the contention over different brands of the intangible contained within itself. That said, the segmentation and disconnection between these elements, following commercial market logics, prevent us from observing how their development has noticeably different effects on each of them. Thus, the promotion of particular “Mediterranean products” generates very negative effects on the socio-ecosystems where they originate—something that, in turn, becomes cause for social inequality and the overexploitation of natural resources. Due to this, part of the Mediterranean Diet is sustained on a social and environmental unsustainability that will not be able to be maintained in the long-term.

In accordance with this market logic, food, landscapes, customs, etc., have been selected as referents of heritage in a process that, equally, disregards everything that does not fit within these parameters and that makes it difficult to consume the image they evoke. Mediterranean landscapes and diet meet in the global space of the tourist who, while consuming both of them at the same time, buys, above all, their image.

In any case, this de-territorialized and de-historized image is continuously reformulated due to tensions between local and global dynamics. On the one hand, Mediterranean gastronomy, on which the diet is based, renovates itself constantly though the transformations (recreations, innovations, and substitutions) that take place within local and regional cuisines. On the other hand, landscapes must continuously adjust to settled processes of socialization and to multi-levelled normative that incorporate local and regional demands linked to the processes that reconfigure them.

## Data Availability

The study did not report any data.
